# A proteomic analysis of liver after ethanol binge in chronically ethanol treated rats

**DOI:** 10.1186/1477-5956-10-29

**Published:** 2012-04-30

**Authors:** Annayya R Aroor, Lowery J Roy, Ricardo J Restrepo, Brian P Mooney, Shivendra D Shukla

**Affiliations:** 1 Department of Medical Pharmacology & Physiology, University of Missouri, Columbia, MO 65212, USA; 2 Proteomics Center, University of Missouri, Columbia, MO 65212, USA; 3 Division of Biochemistry, University of Missouri, Columbia, MO 65212, USA

**Keywords:** Binge ethanol, Chronic ethanol, Liver proteomics

## Abstract

**Background:**

Binge ethanol in rats after chronic ethanol exposure augments necrosis and steatosis in the liver. In this study, two-dimensional gel electrophoresis proteomic profiles of liver of control, chronic ethanol, control-binge, and chronic ethanol- binge were compared.

**Results:**

The proteomic analysis identified changes in protein abundance among the groups. The levels of carbonic anhydrase 3 (CA3) were decreased after chronic ethanol and decreased further after chronic ethanol-binge. Ethanol binge alone in control rats had no effect on this protein suggesting its possible role in increased susceptibility to injury by binge after chonic ethanol treatment. A protein spot, in which both cytosolic isocitrate dehydrogenase (IDH1) and glutamine synthetase (GS) were identified, showed a small decrease after chronic ethanol binge but western blot demonstrated significant decrease only for glutamine synthetase in chronic ethanol treated rats. The level of gluathione S-transferase mu isoform (GSTM1) increased after chronic ethanol but was lower after chronic ethanol-binge compared to chronic ethanol treatment. The protein levels of the basic form of protein disulfide isomerase associated protein 3 (PDIA3) were significantly decreased and the acidic forms were increased after chronic ethanol- binge but not in chronic ethanol treated rats or ethanol binge in control rats. The significant changes in proteome profile in chronic ethanol binge were accompanied by a marked increase in liver injury as evidenced by enhanced steatosis, necrosis, increased 4-hydroxynonenal labeled proteins, CYP2E1 expression, and decreased histone H2AX phosphorylation.

**Conclusions:**

Given the role of CA3, IDH1 and GST in oxidative stress; PDIA3 in protein quality control, apoptosis and DNA repair and decreased glutamine synthetase as a sensitive marker of pericentral liver injury this proteome study of chronic ethanol-binge rat model identifies these proteins for the first time as molecular targets with potential role in progression of liver injury by binge ethanol drinking.

## Background

Alcoholic liver disease is a worldwide health problem [1,2]. The mechanisms that cause progression of liver injury are complex. Healthy liver is resistant to the action of ethanol and most individuals consuming alcohol have steatosis but not steatohepatitis [3]. The progression of steatosis to steatohepatitis has been shown to be dependent on additional factors such as endotoxin, nutritional factors, and other disease states such as hepatitis C viral infection [4-7]. In this regard, binge drinking habit in chronic alcoholics is one of the most important factors contributing to the progression of alcoholic liver injury [8-11]. We have recently developed a chronic ethanol-binge rat model in which short term chronic ethanol treatment for 4 weeks does not result in significant liver injury. When these rats were subjected to 3 episodes of repeat ethanol binge it dramatically amplified liver injury. This rat model mimics findings similar to humans particularly during early alcoholic liver injury [12]. Therefore, it offers an opportunity to determine the effects of binge after chronic ethanol intake and to explore the underlying mechanisms of enhancement of liver injury by binge ethanol.

Recent developments of systems biology approaches including genomics, metabolomics and proteomics offer a better insight into the mechanisms of cellular injury and identification of protein targets without a prior knowledge of the specific underlying molecular pathway [13]. Although gene expression has provided valuable information on transcriptional regulation, significant discrepancy occurs between changes in RNA and protein expression since not all expressed genes are translated into protein products [14]. Two dimensional electrophoresis in combination with mass spectrometry (2DE-MS), has been widely applied for the analyses of protein expression and their post translational modifications [15]. Proteome studies on liver have been useful in elucidating the biomarkers of liver damage and factors that contribute to susceptibility of liver tissue to drug or disease induced liver injury [14-16]. Although, liver proteomic studies have been reported for chronic ethanol treated rats [17-22], proteomics after ethanol binge drinking has not been examined. We have therefore undertaken a 2-dimensional (2D) gel-based proteomics study of liver subjected to binge ethanol administration in vivo.

## Results

### Chronic ethanol, control-binge and chronic ethanol-binge display differential expression of proteins

**Figure 1:**
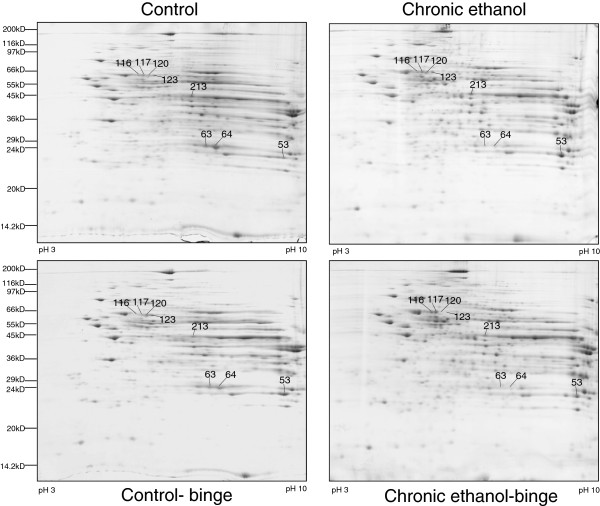
**Composite images of 2D gels of proteins extracted from liver of control, chronic ethanol, control-binge, and chronic ethanol-binge treated rats.** Rats were fed ethanol in liquid diet chronically for 4 weeks and then given three ethanol binges (5 g/kg) at 12 hr intervals. Four hours after the last dose liver samples were frozen in liquid nitrogen and stored for proteome studies. Proteins were extracted by phenol and methanol/ammonium acetate precipitation and 2D electrophoresis and analysis were performed as described under Materials and Methods. Figure 1 represents a composite image of the gel and the proteins that are differentially expressed between the treatment groups and the protein sequenced are shown by spot numbers. Control represents pair –fed animals that were given water for binge control. N = 3 rats for each group.

Rats were divided into four groups: control (pair-fed isocaloric diet), chronic ethanol (4 weeks), control-binge (pair-fed followed by 3 ethanol binge), and chronic ethanol-binge (chronic ethanol 4 weeks followed by 3 binge). Three samples from each group were included for proteome analysis. Figure 1 shows representative 2D gel maps of liver proteins obtained from four groups. A total of 600–725 spots were resolved on these Coomassie Brilliant Blue (CBB) stained 2D gels. Spot volumes (area x intensity) were determined as a measure of protein expression. Spots that showed 1.5 fold increase or decrease were included in the analysis and proteins that are present in appreciable amounts were picked for identification by mass spectrometry (Figure 1).

Eight proteins that were affected by either chronic ethanol or chronic-ethanol binge were selected for identification. These proteins were excised from the gel, digested with trypsin, and subjected initially to MALDI-TOF/TOF MS + MS/MS analysis. Protein identification by MALDI-TOF/TOF was subsequently confirmed by peptide sequences obtained by acquiring multiple LC/MS/MS spectra for each sample. The results are shown in Table 1. Spots 63 & 64 were identified as carbonic anhydrase 3 (CA3) and spots 116, 117, 120 & 123 were identified as isoforms of protein disulfide isomerase associated protein 3 (PDIA3). Spot 213 contained two proteins identified as cytosolic isocitrate dehydrogenase (IDH1) and glutamine synthetase (GS). Spot 53 was identified as glutathione S transferase, mu form (GSTM1).

**Table 1:** Identification of differentially expressed protein spots by mass spectrometry

**Spot No.**	**Identification^a^**	**Accession No.**	**MW (Da)^b^**	**Total Ion Score^c^**	**No. peptides matched**
63	Carbonic anhydrase 3	3137484	29698	206	7
64	Carbonic anhydrase 3	3137484	29698	505	12
213	Cytosolic isocitrate dehydrogenase^d^	6647554	47030	202	10
213	Glutamine synthetase^d^	204349	43001	133	5
53	Glutathione S transferase M1	204503	26127	674	17
116	Protein disulfide isomerase A3	1352384	57044	163	6
117	Protein disulfide isomerase A3	1352384	57044	797	22
120	Protein disulfide isomerase A3	1352384	57044	994	28
123	Protein disulfide isomerase A3	1352384	57044	1294	30

^a^Data from LCMS runs were searched against NCBInr limited to mammals using MASCOT V2.2.

^b^This is the predicted molecular weight based on the translated gene sequence.

^c^The total ion score from the MASCOT report. Individual peptide ion scores >36 have significant identify or homology (*p* < 0.05).

^d^Two proteins were confidently identified in spot number 213. Based on number of peptides matched, isocitrate dehydrogenase is likely the more abundant of the two.

**Figure 2:**
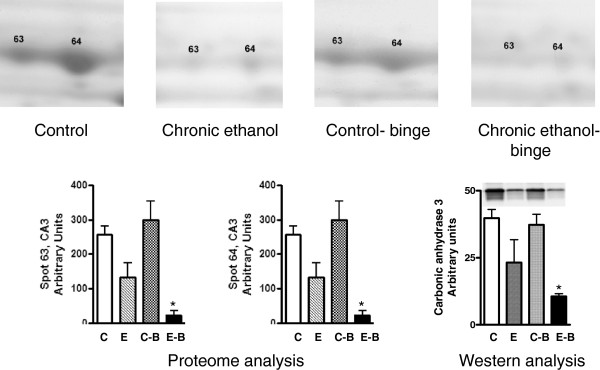
**Quantitative analysis of carbonic anhydrase 3 isoforms from 2D gels and western immunoblot analysis.** Rats were fed ethanol in liquid diet chronically for 4 weeks and then given 3 binge (5 gm/kg) at 12 hr intervals and samples were collected as in Figure 1. The levels of carbonic anhydrase 3 were determined by western immunoblot as described under Materials and Methods. Control represents pair –fed animals that were given water for binge control. Values are mean ± SE (n = 3 rats for each group). Western blot represents a typical experiment. *significant from control group (*p* < 0.05); C: Control (pair fed); E: Chronic ethanol; C-B: Control- ethanol binge; E-B: Chronic-ethanol binge.

**Figure 3:**
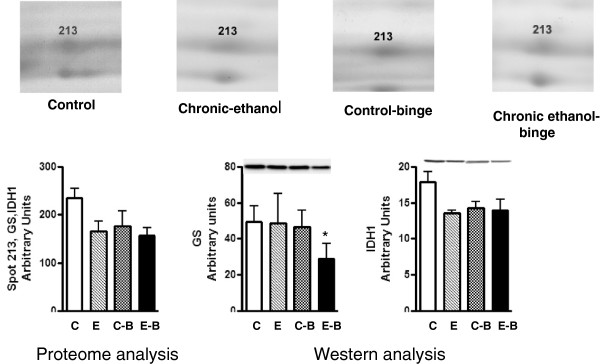
**Quantitative analysis of cytosolic isocitrate dehydrogenase and glutamine synthetase from 2D gels and western immunoblot analysis.** Rats were fed ethanol in liquid diet chronically for 4 weeks and then given 3 binge of ethanol and samples collected as in Figure 1. The levels of isocitrate dehydrogenase and glutamine synthetase were determined by western immunoblot. Control represents pair –fed animals given water for binge control. Values are mean ± SE (n = 4 rats). Western blot represents a typical experiment. *significant from control group (*p* < 0.05); C: Control (pair fed); E: Chronic ethanol; C-B: Control- ethanol binge; E-B: Chronic-ethanol binge.

**Figure 4:**
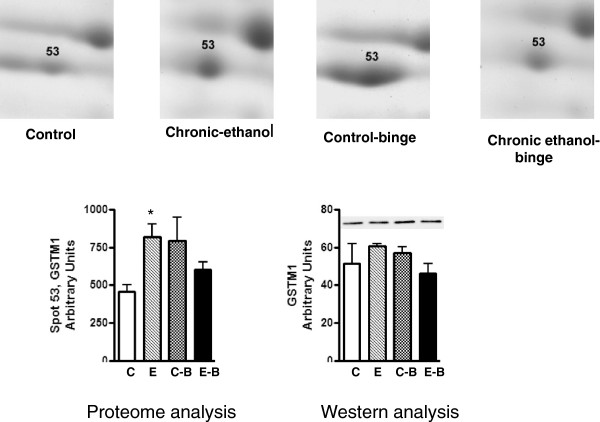
**Quantitative analysis of glutathione S-transferase M1 from 2D gels and western immunoblot analysis.** Rats were fed ethanol in liquid diet chronically for 4 weeks and then given 3 binges before sample collection as detailed in Figure 1. The levels of glutathione transferase mu were determined by western immunoblot. Control represents pair –fed animals that were given water for binge control. Values are mean ± SE (n = 3 rats for each group). Western blot represents a typical experiment. *significant from control group (*p* < 0.05); C: Control (pair fed); E: Chronic ethanol; C-B: Control- ethanol binge; E-B: Chronic-ethanol binge.

**Figure 5:**
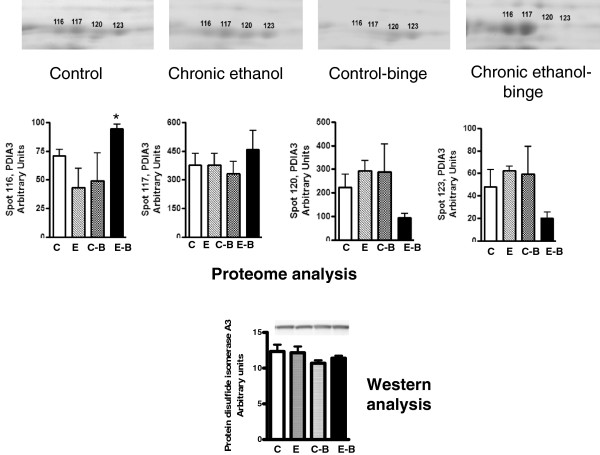
**Quantitative analysis of PDIA3 from 2D gels and western immunoblot analysis.** Sample preparations were similar to that in Figure 1. The upper panel shows 2D proteome profile of the 4 treatment groups (see Figure 1). The spots (# 116, 117, 120 & 123) were quantified and data are presented in the histograms (middle panel). The bottom panel shows western immunoblot using antibody for PDIA3 in the four group samples. Values are mean ± SE (n = 3 rats for each group). Western blot represents a typical experiment. *significant from control group (*p* < 0.05); C: Control (pair fed); E: Chronic ethanol; C-B: Control- ethanol binge; E-B: Chronic-ethanol binge.

**Figure 6:**
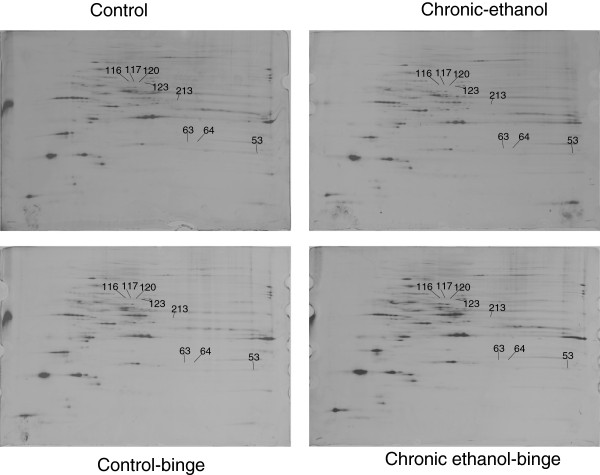
**Composite images of 2D gels of proteins extracted from liver of control, chronic ethanol, control-binge and chronic ethanol-binge treated rats.** Protein samples (as in Figure 1) were extracted by phenol and methanol/ammonium acetate precipitation and 2D electrophoresis and Pro Q staining for phospho-protein analysis were performed as described under Material and Methods. Figure represents pattern of phospho-proteins.

The levels of CA3 decreased after chronic ethanol (Figure 2). Its levels decreased further after chronic ethanol followed by binge . In the control-binge group, CA3 levels were not significantly changed suggesting its role in increased injury by binge ethanol in chronic ethanol treated rats. We have also determined protein levels of CA3 by western immunoblot (Figure 2). The results were in agreement with the proteomic analysis. The spot volume representing combined protein levels of cytosolic isocitrate dehydrogenase/glutamine synthetase in proteome analysis was decreased by about 40% after chronic ethanol–binge (Figure 3). However, the western blot results showed only a small decrease in the protein levels of cytosolic IDH1 in all the treated groups. Interestingly, protein levels of glutamine synthetase decreased in the chronic ethanol-binge group (Figure 3). In the proteome analysis, GSTM1 levels were increased after chronic ethanol and control binge but the levels are lower in chronic ethanol-binge compared to chronic ethanol (Figure 4). However, western immunoblot did not reveal significant increase after chronic ethanol (Figure 4). PDIA3 was identified in multiple forms with shifting of isoelectric point (pI). The protein levels of the more basic forms (spots 120 and 123) were lower in the chronic ethanol binge group whereas the protein levels of the more acidic forms (spots 116 and 117) were higher in the chronic ethanol binge group (Figure 5). The western immunoblot did not reveal any significant change in the expression of PDIA3 in any of the groups (Figure 5). We therefore calculated the sum of the density of all the forms in proteome analysis and observed that the changes in all the three treated groups were not different from the control group (data not shown). These results suggest that, changes in the levels of different isoforms seen after chronic-ethanol binge may represent post translational modifications of the protein. The shift in charge may be due to phosphorylation of PDIA3 forms since phosphorylation of PDIA3 has been shown to occur in vivo and such alterations are associated with a decrease in protein folding function of PDIA3 [23]. To examine this possibility, we performed pro-Q staining that detects phosphorylated forms. Pro Q stain showed significant decrease and increase in several phosphorylated proteins (Figure 6). However, the phosphorylation status of those proteins that showed differential changes in Coomassie blue staining (Table 1) were not altered. It may be mentioned that the quantitative analysis of these phosphorylated spots showed a trend for lowered phosphorylation for basic forms of PDIA3 and increased phosphorylation for acidic form (data not shown).

### Relationship of proteome changes to liver injury during chronic ethanol and chronic ethanol-binge

**Figure 7:**
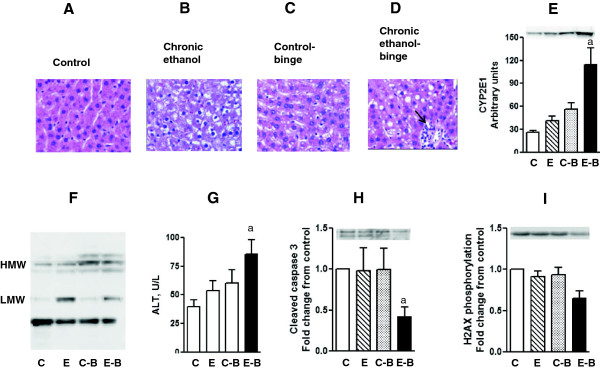
**Parameters of liver injury and histology after chronic and chronic ethanol- three binge.** Rats were fed ethanol in liquid diet chronically for 4 weeks and then given 3 binge (5 gm/kg) at 12 hr intervals. Four hours after the last dose, the levels of serum ALT, and hepatic cleaved caspase-3 were determined as described under Materials and Methods. Sections of liver samples were stained with hematoxylin and eosin. Control represents pair –fed animals and were given water for binge control. Values are mean ± SE (n = 3 rats). Western blot represents a typical experiment. A-D: Hemotoxylin and eosin staining (x 200X); E: CYP2E1 protein expression. F: 4-HNE immunoblot G: Serum ALT; H: hepatic cleaved caspase 3; I: H2AX phosphorylation a: significant from control group (*p* < 0.05); C: Control (pair fed); E: Chronic ethanol; C-B: Control- ethanol binge; E-B: Chronic-ethanol binge. LMW: low molecular weight; HMW: high molecular weight; Small solid arrow represents leukocyte infiltration.

CA3 and IDH1 have been shown to exert protection against oxidative stress and liver injury [24,25]. We examined hepatic steatosis in different groups. The results are shown in Figure 7 A-D. Steatosis was moderate in the chronic ethanol group or control ethanol binge. In contrast, stetaosis was more marked in chronic ethanol binge group. Another parameter of hepatotoxic effects of alcohol is increased expression of CYP2E1 [26,27]. Compared to control, a moderate increase (1.5 to 2 fold) in CYP2E1 protein expression was noted after chronic ethanol and acute three binge whereas a marked increase (about 4 fold) in CYP2E1 was seen after chronic ethanol-binge (Figure 7E). We have also determined the pattern of protein oxidation using antibodies against 4-hydroxynonenal (4-HNE). The increase in 4-HNE is not only an index of cellular oxidative stress but also suggestive of altered function of oxidatively modified proteins [28-30]. A representative western blot is shown in Figure 7F. Chronic ethanol treatment alone caused increases in 4-HNE staining of low molecular weight whereas increases in 4-HNE staining of high molecular weight proteins were seen in the control binge group. In chronic ethanol–binge group there was an increase in 4-HNE staining of low molecular weight as well as of high molecular weight bands demonstrating increased oxidative stress in this group. Consistent with this pattern is the observation that CA3 protein levels decreased in the chronic ethanol group and further decrease in the chronic ethanol-binge (Figure 2). In the chronic ethanol, or control-binge groups a moderate increase in transaminase levels was noted. Its levels increased significantly in the ethanol- binge group suggesting augmentation of liver injury (Figure 7G). Recent studies have shown proapototic role for PDIA3 . This was supported by a decrease in PDIA3 expression and caspase 3 levels [31]. To examine this in our model we determined the expression of cleaved caspase 3 in different groups of ethanol treatment (Figure 7H). Cleaved caspase 3 levels decreased in the chronic ethanol binge. We also examined the relevance of PDIA3. PDIA3 has been implicated in the modulation of histone H2AX phosphorylation. Decreased expression of PDIA3 by siRNA silencing resulted in decreased phosphorylation of H2AX [32]. Since H2AX phosphorylation has been implicated in DNA repair, we therefore examined histone H2AX phosphorylation in the four treatment groups. Histone H2AX phosphorylation was significantly decreased in the chronic-ethanol binge group (Figure 7I).

## Discussion

This is the first proteomic study of global alterations of proteins in liver from chronic ethanol-binge treated animals. The chronic ethanol binge model developed in this laboratory displays a number of features that are seen during human alcoholic liver injury (12). Therefore this model is useful for the investigation of the mechanism of binge induced enhancement of alcoholic liver injury. Using a proteomics approach, we have demonstrated changes in the levels of a number of liver proteins affected differentially by chronic ethanol, control-binge and chronic ethanol-binge. A notable aspect of this study is the identification of these protein targets in a single model. Previously these protein targets have been reported individually in separate studies. The fact that normal liver is resistant to acute ethanol binge [12] as seen in control-binge group in this study, we propose that the increased vulnerability to binge after chronic ethanol treatment and the accompanying alterations in proteins contribute to the amplification of injury.

We find a marked decrease in CA3 in chronic ethanol but not control-binge treated rats. Decrease in CA3 protein level after chronic ethanol administration has been reported [19], but the effects of acute ethanol binge or chronic–ethanol binge were not examined in that study. In another study, chronic ethanol treatment in rats decreased carbonic anhydrase mRNA levels [33] suggesting that the regulation of CA3 occurs at the level of transcription. CA3 is an enzyme that has negligible carbonic anhydrase activity compared to other isoforms of carbonic anhydrase [34]. It has been proposed to have a cytoprotective action against oxidative stress. In NIH/3T3 cells over-expressing CA3, steady state levels of reactive oxygen species are reduced and these cells were protected against hydrogen peroxide induced oxidative stress [35]. CA3 has also been shown to undergo reversible S-thiolation reactions with glutathione and reversible glutathiolation has been proposed to provide protection to the reactive cysteine residues in proteins from damaging oxidative reactions [36]. This assumes significance from studies supporting antioxidant role of CA3. A decrease in CA3 in the present study and augmentation of liver injury as demonstrated by increased transaminase favors the view that it has a protective role. Moreover, lipid peroxidation and oxidative stress has been shown to be increased in an experimental model of chronic ethanol followed by intraperitoneal administration of ethanol binge [37]. In the present study, we observed a marked decrease in CA3 levels in chronic ethanol-binge group that was accompanied by an increase in protein oxidation of low and high molecular weight proteins. There was a predominant increase of protein oxidation of low molecular weight in chronic ethanol group. Levels of oxidation of high molecular weight proteins increased in the control-binge group. In this regard, it may be noted that carbonic anhydrase levels are far lower in humans and they are more susceptible to alcoholic liver injury than rats. Interestingly, female rats have far lower CA3 than male rats and female rats are more susceptible to alcoholic liver injury [38]. Increase in lipid peroxidation was accompanied by increased expression of CYP2E1 and ethanol induced CYP2E1 has been implicated in oxidative stress and steatosis [26,27]. However, the magnitude of increase in the CYP2E1 expression was dramatic in chronic-ethanol binge compared to chronic ethanol or binge alone. These findings suggest that chronic ethanol intake sensitizes liver for ethanol binge induced expression of CYP2E1 to a greater extent than direct effects of ethanol alone.

Isocitrate dehydrogenase 1 (IDH1) is a NADP-dependent enzyme that catalyzes the oxidative decarboxylation of isocitrate to α-ketoglutarate (α-KG) in the cytoplasm/peroxisomes with the concomitant production of NADPH. It is proposed to play an important role in cellular defense against oxidative stress [39]. Treatment of hepatocytes with ethanol or acetaldehyde decreased IDH1 activity, and knock down of IDH1resulted in potentiation of alcohol induced oxidative stress [40]. Decreased activity of IDH1 by oxidative stress has also been reported in liver [41]. In the present study, IDH1 was not significantly decreased after chronic ethanol intake or chronic ethanol-binge.

Glutamine synthetase catalyzes ATP-dependent amidation of glutamate to glutamine. It is responsible for the intrahepatic and inter tissue ammonia elimination/detoxification occurring in the liver [42]. Glutamine synthetase has also been implicated in nucleotide synthesis and amino acid turnover [42]. Glutamine synthetase in normal liver is expressed by a small population of perivenous hepatocytes [42,43]. Decreased expression and activity of glutamine synthetase has been considered to be the sensitive indicator of liver injury and chronic ethanol intake has been shown to result in decreased glutamine synthetase activity [43,44]. Although, glutamine synthetase expression is modestly decreased after chronic ethanol, further decrease occurs after chronic-ethanol binge but not in control-binge. Decreased protein levels of glutamine synthetase in liver may contribute to increased plasma ammonia levels and exaggeration of central nervous system toxicity.

Glutathione S transferase (GST) is a superfamily of enzymes comprising several isoenzymes. The major GST subunits expressed in the adult liver are alpha (A1, A2 and A3) and Mu (M1 and M2). The Pi subunit of GST is only expressed in fetal hepatocytes, and during early stages of hepatocarcinogenesis [45]. GST Pi and M forms are drug metabolizing enzymes and play role in suppressing oxidative stress and reducing the levels of 4-hydroxynonenal [46]. Chronic ethanol exposure has been shown to cause both up- and down- regulation of GST M in vivo and in hepatocytes [46-49]. Accumulation of 4–hydroxynonenal after chronic ethanol treatment is considered to mediate liver injury via lipid peroxidation. Therefore, increased expression of GST that conjugates glutathione to a host of electrophiles [46] may represent an adaptive response induced by chronic ethanol. In this regard, GST M null phenotype has been shown to be associated with increased susceptibility to alcoholic liver injury [50]. In the present study, the expression of GST M1 was marginally increased after chronic ethanol or control ethanol binge, but the levels did not increase after chronic ethanol- binge suggesting compromised GST M1 after chronic ethanol-binge.

An intriguing finding in the present study is the change in the levels of different isoforms of PDIA3 in the chronic ethanol-binge group compared to either chronic ethanol treated or control ethanol binge groups. PDIA3 belongs to the endoplasmic reticular chaperone protein family of protein disulfide isomerases [51]. These more negatively charged isoforms arise by either acetylation or phosphorylation of PDIA3. Recently, increased accumulation of phosphorylated forms of PDIA3 was shown after angiotensin II stimulation of vascular cells [52] or by prolonged fasting in rat liver [23]. Increased carbonylation of liver PDIA3 has been reported after chronic ethanol treatment in rats and this was accompanied by decreased function of the isomerase [53]. PDIA3 is emerging as an important regulator of cell function at multiple levels. It has been implicated in sorting of glycoproteins, quality control of endoplasmic reticular proteins, antigen processing by MHC proteins, control of STAT3 function, induction of apoptosis, and suppression of steatosis [23,31,54,55]. We have observed increased steatosis and suppressed apoptosis after chronic ethanol binge (Figure 7) with concomitant increases in the levels of PDIA3 acidic isoforms (Figure 5). Therefore, these results favor the role of PDIA3 in augmentation of liver injury by ethanol binge. Although suppression of apoptosis is considered as a survival mechanism, such phenomenon under the conditions of toxic insult such as ethanol may be accompanied by disruption of cell repair mechanisms such as repair of damaged DNA. DNA damage and impairment of DNA repair have been reported in animal and human alcoholic liver damage. Relevantly, knockdown of PDIA3 expression impaired phosphorylation of H2AX and DNA repair [32]. We have observed both decreased phosphorylation of H2AX and increase in the levels of acidic isoform of PDIA3 in chronic ethanol-binge group. It is also known that increase in acidic (phosphorylated) isoform of PDIA3 is related to decreased protein folding function of PDIA3 [23]. It is therefore plausible that in chronic ethanol-binge condition changes in PDIA3 disrupt protein folding, decrease H2AX phosphorylation resulting in a compromised DNA repair.

**Figure 8:**
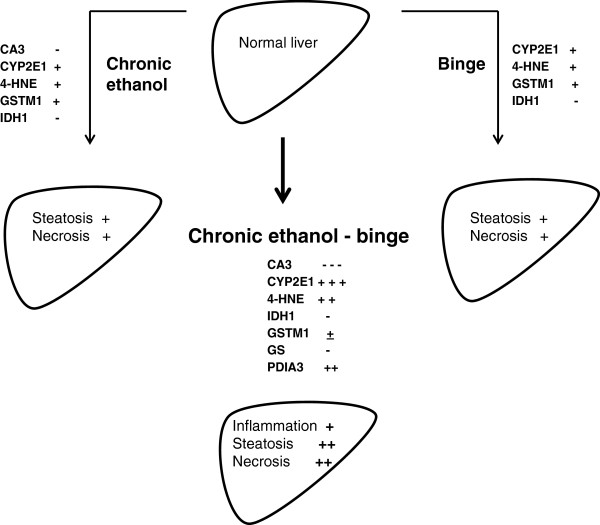
**A flow diagram showing proteome changes and their relationshsip to liver injury by chronic ethanol binge in the rat model.** This schematic figure depicts that binge, and chronic ethanol have their own effects, but chronic ethanol exposure renders liver more susceptible to amplification of injury by binge ethanol. The figure shows changes in the levels of CA3, CYP2E1, 4HNE, PDIA3 and other components after different treatments. Minus (−) and plus (+) symbols indicate the degree of decrease or increase, respectively.

In summary, chronic ethanol and chronic ethanol binge display distinct signature profiles of protein expression. Differential expression of carbonic anhydrase 3, isocitrate dehydrogenase, glutathione S-transferase, glutamine synthetase, and protein disulfide isomerase protein 3 accompany increased susceptibility of chronic ethanol treated rat liver to binge induced injury. The consequences of these proteomic changes on the amplification of alcoholic liver injury by binge ethanol are schematically presented in Figure 8.

## Methods

### Materials

Male Sprague–Dawley rats (250–300 g) were purchased from Harlan Laboratories (Indianapolis, IN). ). The antibodies for carbonic anhydrase 3, protein disulfide isomerase associated protein 3, cytosolic isocitrate dehydrogenase, and glutamine synthetase were purchased from Santa Cruz Biotechnology Inc. (Santa Cruz, CA). Antibodies for histone H2AX and cleaved caspase 3 were purchased form Cell Signaling (Beverly, MA). CYP2E1 antibody was from AbCam Cambridge, MA. Other reagents including protease inhibitor cocktail (Sigma p8340) and β-actin antibody were obtained from the Sigma-Aldrich (St. Louis, MO).

### Animal feeding for chronic ethanol-binge model of alcoholic liver injury

Rats were housed under a 12-h/12-h light/dark cycle and were permitted ad libitum consumption of standard laboratory rat chow. After a one-week adaptation period, the animals were fed Lieber–DeCarli liquid diet (Dyets, Inc., Bethlehem, PA) [20]. Ethanol was progressively introduced into the liquid diet starting at 1.25% (wt./vol.) for day 1, increased to 1.67% (wt./vol.) for day 2, to 2.5% (wt./vol.) for days 3 and 4 and, finally, maintained at 5% (wt./vol.) for 4 weeks. Weight-matched littermates were pair-fed on the same liquid diet, except that the ethanol was replaced by dextrin/maltose (control) to maintain the isocaloric intake in the two groups. Each day, the previous day's ethanol diet intake was measured, and the pair-fed rats were fed same calorie of diet containing dextrin/maltose. After 4 weeks, rats were divided into four groups: control, chronic ethanol, control-binge , chronic-ethanol binge. Chronic ethanol-binge and control-binge groups had three doses (each 5 g/kg body wt) of binge ethanol administered intragastrically every 12 hr. In the control group for chronic ethanol-binge, ethanol was replaced by water. Liver samples were collected 4 hr after the last binge administration (Aroor et al, 2011), and immediately frozen in liquid nitrogen until further use. The animal care and protocol for their use were approved by the University of Missouri Animal Care & Use Committee.

### Two dimensional electrophoresis

Frozen liver samples were pulverized in liquid nitrogen using mortar and pestle. Tris-buffered phenol extraction solution was added to the powdered samples and proteins were extracted using phenol extraction protocol [56]. Protein pellets were resuspended in IEF buffer (8 M Urea, 2 M thiourea, 4% CHAPS, 2% C7BzO, 100 mM DTT, 2.2% 2-HED). Solubilized samples were quantified using the EZQ protocol from Life Technologies (Grand Island, NY). One mg of protein sample was then applied to 24 cm pH 3–10 IPG strips for passive rehydration overnight. The IPG strips were isoelectrically focused on the Protean IEF from Bio-Rad using the following parameters: 250 V for 250Vhr (rapid ramp), 1000 V for 500Vhr (rapid ramp), 8000 V for 2 hours (gradient), 8000 V for 80,000Vhr (rapid ramp), and then hold at 500 V. The IPG strips were then placed on 12% SDS-PAGE gels for the second dimension and ran using the Ettan Dalt 12 at 1 W/gel overnight. After the run, gels were stained with Pro-Q diamond stain and imaged using the Ettan DIGE imager. Gels were then post stained with Coomassie Brilliant Blue (G250) for the total protein and scanned using the UMAX powerlook flatbed scanner. Data were analyzed using Delta 2D system from Decodon after normalization. The identification of spots that showed distinct differences was based on aligning and matching of spots in gels and quantification of matched spots. The spots were also manually inspected to verify the accuracy of matching. We selected a protein spot that showed the least variation between the samples and this spot was used for normalization. All the protein spots chosen were present in all the gels. The results were also analyzed based on normalization to total spot volume and the pattern was similar to data analysis based on normalization to the protein spot with the least variation among the gels. For phosphoprotein analysis, the values were normalized for total protein.

### Analysis of proteins by mass spectrometry

Spots were excised from the gel and subjected to trypsin digest using a standard in-gel digestion protocol [57]. The extracted peptides were lyophilized and resuspended in 3 μl of 1% formic acid. A 0.5 μl aliquot of the resulting peptides was mixed 1:1 with CHCA matrix (5 mg/mL in 60% ACN, 0.3% TFA, 10 mM ammonium phosphate) and spotted onto the MALDI target. The instrument (Applied Biosystems 470 Proteomics Analyzer) was operated in positive ion mode and spectra were acquired over a mass range of 700 to 4000 Da. Peptide calibration standards (4700 calibration mix, Applied Biosystems) were used to calibrate the instrument in MS mode using six peptides of known mass. Calibration was achieved by the “plate model and default” mode for MS of six external calibrant spots. Additionally, internal (i.e. within the sample spot) calibration was achieved using trypsin autolysis peptides (where present). Note: although trypsin autolysis ions are useful for obtaining the best mass accuracy, these ions are automatically excluded from the database search conducted using the GPS Explorer.

Following an MS scan of each sample the 8 most abundant peptides were picked automatically for tandem MS (MSMS – peptide fragmentation) acquisition. Following this a database search was performed using V3.6 of GPS Explorer. Ions with s/n ratios >20 (excluding trypsin autolysis) up to a max of 125 per spectrum were submitted for searches against mammal entries in the NCBInr database (last updated September 9^th^ 2010) using the “combined MS + MSMS” function of the GPS Explorer software. The GPS Explorer software is integrated with the MASCOT V2.2 (http://www.matrixscience.com). Search parameters allowed: 1 trypsin miss-cleavage and the following modifications to peptides: carbamidomethyl cysteines, and methionine oxidation. MS mass tolerance was 100 ppm, MSMS mass tolerance was 0.2 Da.

Liquid Chromatography Tandem Mass Spectrometry analysis: Digests were diluted to 5 μl with 1% formic acid and transferred to an autosampler vial and placed in the autosampler which was maintained at 4°C. A portion of the sample (3 μL) was loaded onto a 150 mm C18 CHIP (Agilent Technologies cat# G4240-62002). Peptides were separated and eluted from the analytical column with a continuous gradient of acetonitrile from 3 to 45% (in 0.1% formic acid) over 8 minutes. Following an MS scan of the eluting peptides, each cycle, the five most abundant peptides were subjected to peptide fragmentation (MSMS). Data across 17 minutes were collected. MSMS data were extracted and a peak list file (mgf – mascot generic format file) was generated using the qualitative analysis software. A search of NCBInr-limited to mammals was conducted using a local copy of MASCOT server V2.2. We selected only eight proteins by proteome analysis. The use of Coomassie blue staining and use of broader pH range might have resulted in the detection of lower number of spots. Our objective was to cover wide range of proteins and obtain adequate amount of protein for mass spectrometry analysis. Therefore, we have adopted this protocol. The limited number spot difference has also been reported for other proteome studies in chronic ethanol administration and endotoxin induced liver injury [19,58].

### Statistical analysis

Data are expressed as means ± S.E.M. Statistical significance of the differences between means was assessed by Student's *t* test. A probability of less than 5% was considered significant (*P* < 0.05).

## Abbreviations

ALT = Alanine transaminase; CA3 = Carbonic anhydrase 3; CBB = Coomassie brilliant blue; CYP2E1 = Cytochrome P 450 2E1; GS = Glutamine synthetase; H2AX = Histone H2A X; HNE = 4-hydroxynonenal; GSTM1 = Glutathione S-transferase, mu isoform; MS = Mass spectrometry; IDH1 = Cytosolic isocitarate dehydrogenase; PDIA3 = Protein disulfide isomerase associated protein 3.

## Competing interests

The authors declare that they have no competing interests.

## Authors’ contributions

ARA developed chronic ethanol binge animal model, conducted experiments, performed western immuno blots, did analysis of the results, and wrote the manuscript. LJR performed protein extraction, two dimensional electrophoresis and proteomic data handling. RJR conducted western blots, analysed the data and helped in manuscript writing. MBP did the MALDI-TOF-MS/MS analysis, contributed to the technical part on proteome experiments and supervised proteome experiments. SDS supervised the planning & design of experiments, analysis & interpretation of the results, and preparation of the manuscript. All authors read and approved the final manuscript.
